# 6-Oxo-1,6-dihydro­pyridazine-3-carbaldehyde monohydrate

**DOI:** 10.1107/S1600536812031674

**Published:** 2012-07-18

**Authors:** Lei Wang

**Affiliations:** a School of Chemistry and Chemical Engineering, Linyi University, Linyi 276005, People’s Republic of China

## Abstract

In the title hydrate, C_5_H_4_N_2_O_2_·H_2_O, the pyridazine ring is essentially planar, with an r.m.s. deviation of 0.0025 Å. In the crystal, O—H⋯O and N—H⋯O hydrogen bonds link the mol­ecules into a one-dimensional chain.

## Related literature

For the biological functions of pyridazine and its derivatives, see: Heinisch & Kopelent (1992 ▶). For bond lengths and angles in related compounds, see: Sarkhel & Desiraju (2004 ▶).
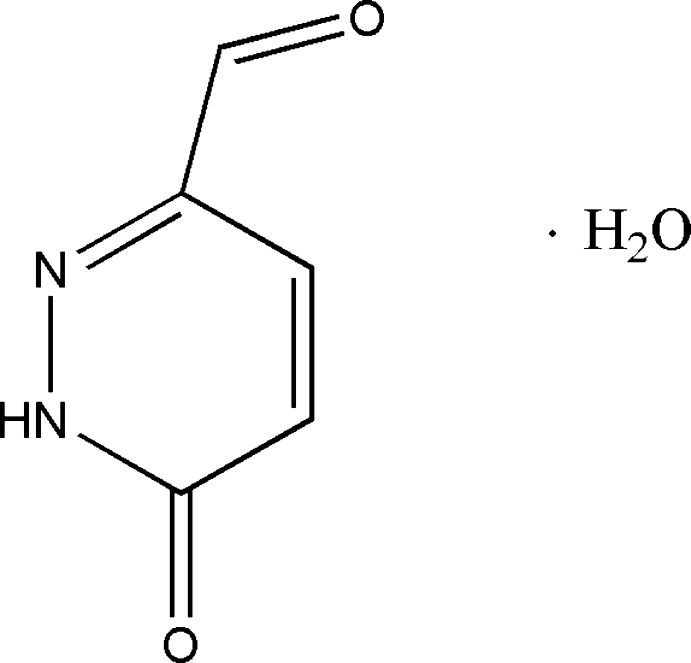


## Experimental

### 

#### Crystal data

C_5_H_4_N_2_O_2_·H_2_O
*M* *_r_* = 142.12
Monoclinic, 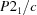

*a* = 8.978 (2) Å
*b* = 6.4150 (16) Å
*c* = 11.354 (3) Å
β = 101.696 (3)°
*V* = 640.4 (3) Å^3^
*Z* = 4
Mo *K*α radiation
μ = 0.12 mm^−1^
*T* = 296 K
0.20 × 0.18 × 0.11 mm

#### Data collection

Bruker SMART CCD area-detector diffractometer
Absorption correction: multi-scan (*SADABS*; Sheldrick, 1996 ▶) *T*_min_ = 0.976, *T*_max_ = 0.987
3981 measured reflections
1190 independent reflections
862 reflections with *I* > 2σ(*I*)
*R*_int_ = 0.024

#### Refinement

*R*[*F* ^2^ > 2σ(*F* ^2^)] = 0.051
*wR*(*F* ^2^) = 0.159
*S* = 1.06
1190 reflections
92 parameters
H-atom parameters constrained
Δρ_max_ = 0.30 e Å^−3^
Δρ_min_ = −0.21 e Å^−3^

### 

Data collection: *SMART* (Bruker, 2004 ▶); cell refinement: *SMART*; data reduction: *SAINT* (Bruker, 2004 ▶); program(s) used to solve structure: *SHELXS97* (Sheldrick, 2008 ▶); program(s) used to refine structure: *SHELXL97* (Sheldrick, 2008 ▶); molecular graphics: *SHELXTL* (Sheldrick, 2008 ▶); software used to prepare material for publication: *SHELXTL*.

## Supplementary Material

Crystal structure: contains datablock(s) global, I. DOI: 10.1107/S1600536812031674/jj2141sup1.cif

Structure factors: contains datablock(s) I. DOI: 10.1107/S1600536812031674/jj2141Isup2.hkl

Supplementary material file. DOI: 10.1107/S1600536812031674/jj2141Isup3.cml

Additional supplementary materials:  crystallographic information; 3D view; checkCIF report

## Figures and Tables

**Table 1:** Hydrogen-bond geometry (Å, °)

*D*—H⋯*A*	*D*—H	H⋯*A*	*D*⋯*A*	*D*—H⋯*A*
N2—H1⋯O3^i^	0.86	1.91	2.745 (3)	165
O3—H1*W*⋯O2^ii^	0.80	2.00	2.794 (3)	173
O3—H2*W*⋯O2^iii^	0.78	2.01	2.790 (2)	172

Symmetry codes: (i) 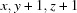; (ii) 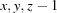; (iii) 
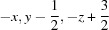
.

**Fig. 1.**
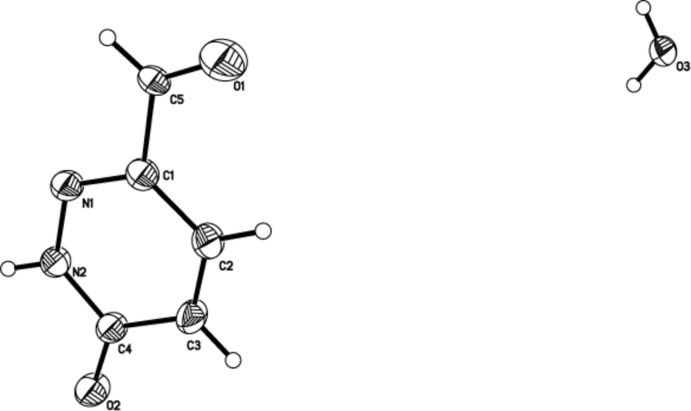
The molecular structure of (I), with atom labels and 50% probability displacement ellipsoids for non-H atoms.

**Fig. 2.**
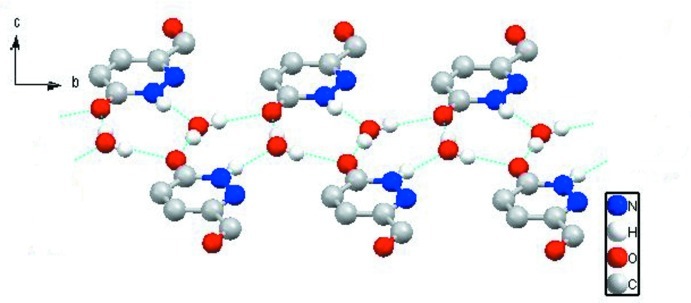
The molecular packing for (I) viewed along the a axis. O—H···O and N—H···O hydrogen bonds are shown by dashed lines linking the molecules into a one-dimensional chain.

## supplementary crystallographic information

### Comment

Pyridazine derivatives are a family of very important compounds due to their antiinflammatory, antimicrobial, insecticidal and herbicidal activities. Compounds with different activity can be obtained when different groups are introduced into pyridazine structures (Heinisch & Kopelent, 1992). Hydrogen bonds have been shown to play important roles in the physical, chemical, and biological properties of many chemical processes. In the title compound, (I), N—H..O and O—H···O hydrogen bonds have been observed. The title compound, C_5_H_4_N_2_O_2_.H_2_O, crystallizes with an organic molecule and a water molecule in the asymmetric unit (Fig. 1). The pyridazine ring is essential planar, with an r.m.s. deviation of 0.0025 Å. The O2, C5 and O1 substituents are coplanar with the mean plane of the pryidazine ring [displacements = 0.0364, -0.0058 and -0.0146 Å, respectively]. Bond lengths and angles are within normal ranges (Sarkhel & Desiraju, 2004). In the crystal, O—H···O and N—H···O hydrogen bonds (Table 1) link the molecules into a one-dimensional chain (Fig. 2).

### Experimental

To a solid of 3-Chloro-6-methylpyridazine (5 mmol) in dry dioxane was added SeO_2_ (1.5 g). The mixture was stirred for 6 h at the reflux temperature of dioxane. After evaporation of the solvent, the residue was purified by column chromatography on silica gel (ethyl acetate) to afford the title compound as a light yellow solid (497 mg, yield 70%). The title compound was recrystallized from methanol at room temperature to give the desired crystals suitable for single-crystal X-ray diffraction.

### Refinement

H1W and H2W were located by a difference map and refined isotropically. All of the remaining H atoms were positioned geometrically and treated as riding, with C—H bonding lengths constrained to 0.93 Å (aromatic CH) or 0.97 Å (methylene CH_2_), and with *U*_iso_(H) = 1.2Ueq(C) or 1.5Ueq(methylene C).

### Crystal data

**Table:**

C_5_H_4_N_2_O_2_·H_2_O	*F*(000) = 296
*M**_r_* = 142.12	*D*_x_ = 1.474 Mg m^−^^3^
Monoclinic, *P*2_1_/*c*	Mo *K*α radiation, λ = 0.71073 Å
Hall symbol: -P 2ybc	Cell parameters from 1099 reflections
*a* = 8.978 (2) Å	θ = 3.7–25.6°
*b* = 6.4150 (16) Å	µ = 0.12 mm^−^^1^
*c* = 11.354 (3) Å	*T* = 296 K
β = 101.696 (3)°	Block, colourless
*V* = 640.4 (3) Å^3^	0.20 × 0.18 × 0.11 mm
*Z* = 4	

### Data collection

**Table:**

Bruker SMART CCD area-detector diffractometer	1190 independent reflections
Radiation source: fine-focus sealed tube	862 reflections with *I* > 2σ(*I*)
Graphite monochromator	*R*_int_ = 0.024
φ and ω scans	θ_max_ = 25.5°, θ_min_ = 2.3°
Absorption correction: multi-scan (*SADABS*; Sheldrick, 1996)	*h* = −10→10
*T*_min_ = 0.976, *T*_max_ = 0.987	*k* = −7→7
3981 measured reflections	*l* = −13→13

### Refinement

**Table:**

Refinement on *F*^2^	Secondary atom site location: difference Fourier map
Least-squares matrix: full	Hydrogen site location: inferred from neighbouring sites
*R*[*F*^2^ > 2σ(*F*^2^)] = 0.051	H-atom parameters constrained
*wR*(*F*^2^) = 0.159	*w* = 1/[σ^2^(*F*_o_^2^) + (0.0736*P*)^2^ + 0.3841*P*] where *P* = (*F*_o_^2^ + 2*F*_c_^2^)/3
*S* = 1.06	(Δ/σ)_max_ < 0.001
1190 reflections	Δρ_max_ = 0.30 e Å^−^^3^
92 parameters	Δρ_min_ = −0.21 e Å^−^^3^
0 restraints	Extinction correction: *SHELXL97* (Sheldrick, 2008), Fc^*^=kFc[1+0.001xFc^2^λ^3^/sin(2θ)]^-1/4^
Primary atom site location: structure-invariant direct methods	Extinction coefficient: 0.009 (5)

### Special details

**Table:**

Geometry. All e.s.d.'s (except the e.s.d. in the dihedral angle between two l.s. planes) are estimated using the full covariance matrix. The cell e.s.d.'s are taken into account individually in the estimation of e.s.d.'s in distances, angles and torsion angles; correlations between e.s.d.'s in cell parameters are only used when they are defined by crystal symmetry. An approximate (isotropic) treatment of cell e.s.d.'s is used for estimating e.s.d.'s involving l.s. planes.
Refinement. Refinement of *F*^2^ against ALL reflections. The weighted *R*-factor *wR* and goodness of fit *S* are based on *F*^2^, conventional *R*-factors *R* are based on *F*, with *F* set to zero for negative *F*^2^. The threshold expression of *F*^2^ > σ(*F*^2^) is used only for calculating *R*-factors(gt) *etc.* and is not relevant to the choice of reflections for refinement. *R*-factors based on *F*^2^ are statistically about twice as large as those based on *F*, and *R*-factors based on ALL data will be even larger.

### Fractional atomic coordinates and isotropic or equivalent isotropic displacement parameters (Å^2^)

**Table:**

	*x*	*y*	*z*	*U*_iso_*/*U*_eq_	
N1	0.2572 (2)	1.0090 (3)	0.98272 (18)	0.0477 (6)	
N2	0.1866 (2)	0.9050 (3)	1.05760 (18)	0.0452 (6)	
H1	0.1462	0.9788	1.1061	0.054*	
O1	0.4654 (3)	0.9374 (4)	0.7609 (2)	0.0853 (8)	
O2	0.0999 (2)	0.6205 (3)	1.13746 (18)	0.0611 (6)	
O3	0.0862 (3)	0.2000 (3)	0.19846 (18)	0.0672 (7)	
H1W	0.0911	0.3173	0.1755	0.101*	
H2W	0.0389	0.1858	0.2485	0.101*	
C1	0.3210 (3)	0.8957 (4)	0.9111 (2)	0.0456 (7)	
C2	0.3184 (3)	0.6758 (4)	0.9111 (2)	0.0524 (7)	
H2	0.3661	0.6012	0.8590	0.063*	
C3	0.2468 (3)	0.5760 (4)	0.9866 (2)	0.0528 (7)	
H3	0.2453	0.4311	0.9885	0.063*	
C4	0.1722 (3)	0.6946 (4)	1.0650 (2)	0.0461 (7)	
C5	0.4022 (3)	1.0224 (4)	0.8288 (2)	0.0410 (6)	
H5	0.4017	1.1673	0.8323	0.049*	

### Atomic displacement parameters (Å^2^)

**Table:**

	*U*^11^	*U*^22^	*U*^33^	*U*^12^	*U*^13^	*U*^23^
N1	0.0609 (14)	0.0400 (12)	0.0494 (12)	−0.0020 (10)	0.0286 (11)	0.0013 (9)
N2	0.0585 (13)	0.0379 (12)	0.0486 (12)	−0.0005 (10)	0.0332 (10)	−0.0021 (9)
O1	0.1000 (18)	0.0802 (17)	0.0927 (16)	−0.0064 (14)	0.0598 (15)	0.0048 (14)
O2	0.0840 (14)	0.0454 (11)	0.0703 (13)	−0.0035 (10)	0.0541 (11)	0.0018 (9)
O3	0.1018 (17)	0.0420 (11)	0.0768 (14)	0.0021 (10)	0.0626 (13)	−0.0010 (9)
C1	0.0521 (15)	0.0438 (15)	0.0459 (14)	−0.0015 (12)	0.0218 (12)	0.0003 (11)
C2	0.0645 (17)	0.0477 (16)	0.0545 (16)	0.0049 (13)	0.0342 (14)	−0.0041 (12)
C3	0.0706 (18)	0.0361 (14)	0.0623 (16)	0.0006 (13)	0.0383 (14)	−0.0041 (12)
C4	0.0567 (16)	0.0379 (15)	0.0508 (14)	0.0009 (12)	0.0276 (12)	0.0012 (11)
C5	0.0474 (13)	0.0441 (14)	0.0389 (12)	−0.0028 (11)	0.0260 (11)	0.0014 (10)

### Geometric parameters (Å, º)

**Table:**

N1—C1	1.306 (3)	C1—C2	1.410 (4)
N1—N2	1.337 (3)	C1—C5	1.531 (3)
N2—C4	1.360 (3)	C2—C3	1.335 (4)
N2—H1	0.8600	C2—H2	0.9300
O1—C5	1.179 (3)	C3—C4	1.435 (3)
O2—C4	1.241 (3)	C3—H3	0.9300
O3—H1W	0.8002	C5—H5	0.9300
O3—H2W	0.7808		
			
C1—N1—N2	116.3 (2)	C1—C2—H2	120.3
N1—N2—C4	126.68 (19)	C2—C3—C4	119.3 (2)
N1—N2—H1	116.7	C2—C3—H3	120.3
C4—N2—H1	116.7	C4—C3—H3	120.3
H1W—O3—H2W	114.8	O2—C4—N2	119.3 (2)
N1—C1—C2	123.2 (2)	O2—C4—C3	125.5 (2)
N1—C1—C5	114.1 (2)	N2—C4—C3	115.2 (2)
C2—C1—C5	122.8 (2)	O1—C5—C1	120.3 (3)
C3—C2—C1	119.3 (2)	O1—C5—H5	119.8
C3—C2—H2	120.3	C1—C5—H5	119.8
			
C1—N1—N2—C4	−1.4 (4)	N1—N2—C4—O2	−178.3 (2)
N2—N1—C1—C2	−0.4 (4)	N1—N2—C4—C3	2.7 (4)
N2—N1—C1—C5	−179.1 (2)	C2—C3—C4—O2	178.7 (3)
N1—C1—C2—C3	0.5 (5)	C2—C3—C4—N2	−2.3 (4)
C5—C1—C2—C3	179.1 (2)	N1—C1—C5—O1	179.3 (3)
C1—C2—C3—C4	0.9 (4)	C2—C1—C5—O1	0.5 (4)

### Hydrogen-bond geometry (Å, º)

**Table:**

*D*—H···*A*	*D*—H	H···*A*	*D*···*A*	*D*—H···*A*
N2—H1···O3^i^	0.86	1.91	2.745 (3)	165
O3—H1*W*···O2^ii^	0.80	2.00	2.794 (3)	173
O3—H2*W*···O2^iii^	0.78	2.01	2.790 (2)	172

Symmetry codes: (i) *x*, *y*+1, *z*+1; (ii) *x*, *y*, *z*−1; (iii) −*x*, *y*−1/2, −*z*+3/2.
